# DECREASED FREQUENCY OF SMALL TALK DURING COVID-19 PANDEMIC AND MENTAL HEALTH: LONGITUDINAL SURVEYS IN JAPAN

**DOI:** 10.1093/geroni/igac059.2632

**Published:** 2022-12-20

**Authors:** Hiroshi Murayama, Ikuko Sugawara

**Affiliations:** Tokyo Metropolitan Institute of Gerontology, Tokyo, Tokyo, Japan; Bunri University of Hospitality, Suginami-ku, Tokyo, Japan

## Abstract

The coronavirus disease 2019 (COVID-19) has drastically reduced opportunities for small talk. As small talk involves socializing, such deprivation can be stressful. This study examined the association between the change in frequency of small talk before and during the pandemic and the mental health of middle-aged and older people. We conducted web-based longitudinal questionnaire surveys from March to May 2020 and from September to October 2021 among members of a Japanese social networking service. We analyzed 867 responses of people who participated in both surveys (mean age, 68.0 ± 8.0 years; men, 68.1%). This study was approved by the Research Ethics Committee of University of Tokyo. Change in small talk frequency before and during the pandemic was assessed using a single item, “Have the opportunities for small talk (including face-to-face, phone, video call, etc.) with someone other than cohabiting family members changed?” We divided the responses into “increase,” “no change,” and “decrease.” Mental health outcomes included psychological well-being and loneliness. A total of 57.0%, 34.4%, and 8.7% reported “decrease,” “no change,” and “increase,” respectively, from before to during the pandemic. After adjusting for potential covariates, multiple regression analyses showed that people who felt their small talk frequency decreased during the pandemic compared to pre-pandemic period had lower psychological well-being and greater loneliness than those who did not. We observed no interaction between change in small talk frequency and age/sex. Our study quantitatively revealed the importance of small talk during the pandemic in maintaining mental health.

